# Knowledge, practice, and risk of exposure of abattoir workers to *Mycobacterium* spp. in abattoirs and non-abattoir environments in Gauteng province, South Africa

**DOI:** 10.4102/ojvr.v93i1.2250

**Published:** 2026-03-18

**Authors:** Vuyokazi E. Mareledwane, Tiny M. Hlokwe, Yusuf B. Ngoshe, Abiodun A. Adesiyun

**Affiliations:** 1 Department of Science and Innovation, Technology Innovation Agency, Pretoria, South Africa; 2 Department of Bacteriology, Agricultural Research Council, Pretoria, South Africa; 3 Department of Production Animal Studies, Faculty of Veterinary Science, University of Pretoria, Pretoria, South Africa; 4 School of Veterinary Medicine, Faculty of Medical Sciences, University of the West Indies, St. Augustine, Trinidad and Tobago

**Keywords:** *Mycobacterium* spp., zoonotic tuberculosis, exposure risk, abattoir workers, knowledge

## Abstract

Zoonotic tuberculosis (TB), caused by *Mycobacterium bovis*, a member of the *Mycobacterium tuberculosis* complex, remains a global public health hazard. Information based on knowledge, practices, and associated risks among high-risk occupations, such as veterinarians and abattoir workers, is crucial. A close-end questionnaire was used to interview 103 abattoir workers during sampling visits, and data on their knowledge, practices, and potential exposure to *Mycobacterium* spp. were collected. More males (87/103, 84.47%) participated in the study, with the majority (83/103, 80.58%) working in the abattoirs’ slaughter and meat processing sections. A high proportion (88.35%) of participants were knowledgeable about TB. A relatively low proportion of abattoir workers were aware that they could transmit TB to animals (44.6%) and that they could acquire the disease from animals (45.7%), suggesting that they may know about the disease but little about its mode of transmission. To reduce exposure to infectious bacteria, personal protective equipment (PPE) was worn when handling carcasses at abattoirs, with 95.15% (95% confidence interval [CI]: 90.92–99.37) reporting its use. Non-abattoir potential exposure to *Mycobacterium* spp. among abattoir workers included the consumption of undercooked meat (10.68%; 95% CI: 4.61–16.75), slaughtering of animals at home (47.57%; 95% CI: 37.76–57.38), and consuming raw milk (25.24%; 95% CI: 16.71–33.77). It is therefore not enough for abattoir workers to know about bovine TB; they must also understand other aspects of the disease, particularly its transmission.

**Contribution:**

Our findings revealed a high proportion of workers following good PPE donning practices when handling carcasses, thereby minimising the transmission of zoonotic diseases such as TB. Consumption of raw milk and undercooked meat are significant risks associated with the transmission of zoonotic TB. Therefore, to reduce the risks and improve the overall well-being, awareness programmes regarding control and prevention are crucial.

## Introduction

Human and animal tuberculosis (TB) are primarily caused by *Mycobacterium tuberculosis* and *Mycobacterium bovis*, respectively, which are members of the *Mycobacterium tuberculosis* complex (MTBC) with genetic similarities (Rogall et al. 1990). Zoonotic TB is caused by *M. bovis* (Weldegebriel et al. 2025). Consequently, it has been reported that out of 10 million people diagnosed with active TB, approximately 140 000 of those cases are estimated to be zoonotic TB cases, with 11 400 deaths recorded worldwide, and the highest incidence recorded on the African continent (Kock et al. 2021). No accurate data are available on zoonotic TB, so it is impossible to estimate the actual disease burden, indicating that cases of humans contracting zoonotic TB might be higher than estimated. Additionally, published data usually do not represent zoonotic TB nationally but typically focus on selected groups (Olea-Popelka et al. 2017). Furthermore, published reports document increased risk of transmission in TB-endemic areas where humans, such as veterinarians and abattoir workers, come into direct contact with infected animals (Cosivi et al. 1998) or with animal products, such as unpasteurised milk (Michel et al. 2015).

Overall, it has been advocated by Adesokan et al. (2013) that the knowledge and practices regarding zoonotic TB could be better investigated in occupationally exposed individuals. Hence, this study analysed the knowledge and practice regarding zoonotic TB by red meat abattoir workers in the Gauteng province of South Africa. Additionally, we assessed their risk of exposure to *Mycobacterium* spp., including the MTBC, the bacterial agents causing TB, at work, home and outdoor environments.

## Research methods and design

### Study area

**FIGURE 1:**
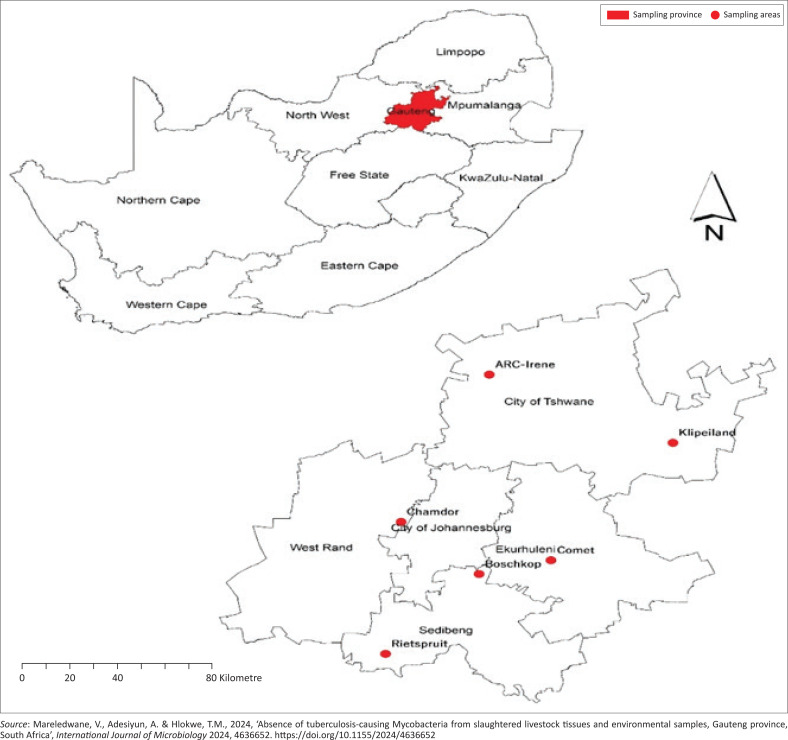
Map showing the location of the red meat abattoirs in Gauteng province where the questionnaires were administered.

The study was conducted at six red meat abattoirs in Gauteng province, South Africa, located in the following districts: Irene and Klipeiland abattoirs are located in the City of Tshwane district; Chamdor abattoir is located in the West Rand district; Comet abattoir is situated in the Ekurhuleni district; Bochkop and Rietspruit abattoirs are located in the Sedibeng district (Figure 1). The current questionnaire-administered study was conducted after an initial study was conducted at 19 abattoirs, where blood samples from slaughtered cattle were collected to determine the presence of cell-mediated immunity (CMI) to *Mycobacterium* spp. as measured by the gamma-interferon assay, as documented in our two previous studies (Mareledwane et al. 2021; Mareledwane, Adesiyun & Hlokwe 2024). Briefly, the investigators obtained a list of functional red meat abattoirs (mono- and multi-species) located across Gauteng province from the Gauteng Department of Agriculture and Rural Development (GDARD). Based on the information provided for each abattoir, which included the type and number of livestock slaughtered daily, location, and their facilities, a total of 19 abattoirs were randomly selected, comprising 16 high-throughput (HT) and three low-throughput (LT) abattoirs for the study. These prior studies included collecting samples from the same 19 abattoirs to determine the prevalence of brucellosis, leptospirosis, Q fever, toxoplasmosis, and TB (Dogonyaro et al., 2020; Kolo et al. 2019; Mangena et al. 2023; Mareledwane et al. 2021).

The six abattoirs used in this study were therefore randomly selected from the 19. Overall, the six abattoirs selected for the study comprised three LT abattoirs, each with approximately 6–50 workers, and three HT abattoirs, each with approximately 500–1500 workers. The status of the six abattoirs used in our study was as follows: Irene (LT, 6 workers), Klipeiland (LT, 6 workers), Boschkop (LT, 10 workers), Chamdor (HT, 30 workers), Comet (HT, 25 workers), and Rietspruit (HT, 26 workers). Therefore, 103 workers participated in the study. The sample size estimation for the follow-up questionnaire study was previously described (Kolo et al. 2024).

### Study design and questionnaire

A close-end questionnaire was prepared and administered to 103 abattoir workers from six different abattoirs between March 2018 and May 2018. The questionnaire was pre-tested in 10 workers at each of the two abattoirs (HT and LT), and all the necessary changes were made before the study commenced. We selected workers from both high and low-throughput abattoirs. Participating abattoir workers were selected through random sampling, and their willingness to participate. The questionnaire was developed to assess the abattoir workers’ knowledge and practices that could predispose them to TB in both work and outside work environments. Verbal and written consent was sought before the interviews were conducted. For the workers who agreed to participate in the study, a briefing session was held at the selected abattoirs to explain the objectives of the study and to provide an explanation of each question in the questionnaire to be administered.

### Statistical analysis

Data were analysed using the Statistical Package Stata 14 (StataCorp, 2015. Stata Statistical Software: Release *14*. College Station, Texas: StataCorp LP, United States) for descriptive analysis. Tables and bar charts were constructed using Microsoft^®^ Excel 2010. Values of *p* < 0.05 were regarded as statistically significant. Data were presented as frequencies and percentages. The data from the study were subjected to univariable analysis to estimate 95% confidence intervals (CIs).

### Ethical considerations

Ethical clearance to conduct this study was obtained from the University of Pretoria Faculty of Health Sciences Research Ethics Committee (No. 519/2017), University of Pretoria Animal Ethics Committee (No. V104-17), Agricultural Research Council-Onderstepoort Veterinary Research Animal Ethics Committee (No. AEC 12.16) and the Department of Agriculture, Land Reforms and Rural Development (DALRRD) (No. 12/11/1/1/6).

## Results

### Socio-demographic information

A total of 103 abattoir workers participated in the study, including management (*n* = 17) and general abattoir workers (*n* = 86). There were more male (84.47%, *n* = 87/103) than female respondents (15.53%, *n* = 16/103). Most respondents (41.75%, *n* = 43/103) were between 26 years and 35 years of age. Also, many respondents (80.58%, *n* = 83/103) worked in the abattoirs’ slaughter and meat processing sections, rather than in the loader or transporter section (Table 1).

**TABLE 1:** Socio-demographic features of the survey respondents from the different districts in the Gauteng province (*N* = 103).

Variable	Category	Frequency (*n*)	Frequency (%)
Age group (years)	18–25	14	13.59
	26–35	43	41.75
	36–50	33	32.04
	51–60	13	12.62
Gender	Female	16	15.53
	Male	87	84.47
Marital status	Single	61	59.22
	Married	42	40.78
Occupation (Job)	Hide processor	1	0.97
	Loader	1	0.97
	Management	17	16.50
	Slaughter and processing	83	80.58
	Transporter	1	0.97
How long have you worked at the abattoir? (years)	1	21	20.39
	2	17	16.50
	3	64	62.14
	4	1	0.97

### Knowledge of tuberculosis among abattoir workers

Knowledge of TB among abattoir workers was high at a frequency of 88.35% (95% CI: 82.04–94.65). When asked about the zoonotic implications, more than 45% (95% CI: 35.28–56.02) of abattoir workers knew that they could be infected with *M. bovis* from infected animals, while 44.6% (95% CI: 34.22–54.91) were aware that they could transmit TB to animals if they are infected with the TB-causing pathogens. A low percentage of the respondents, 7% (95% CI: 2.09–13.13), were previously diagnosed with TB (Table 2).

**TABLE 2:** Participants’ responses to questions about their knowledge of tuberculosis and its transmission.

Variable	Category	%	Frequency		95% CI
			*n*	*N*	
Do you know what TB is?	Yes	88.35	91	103	82.04–94.65
Have you ever been sick with TB?	Yes	7.60	7	92	2.09–13.13
Has any member of your family been sick with TB before?	Yes	18.20	17	93	10.28–26.28
Do you think you can get TB from animals?	Yes	45.70	42	92	35.28–56.02
Do you think you can infect animals with TB?	Yes	44.60	41	92	34.22–54.91

TB, tuberculosis; CI, confidence interval.

### Practices that potentially promote the transmission of TB-causing mycobacteria between abattoir workers and animals

Practices that could promote the transmission of TB between animals and abattoir workers were identified and highlighted in Table 3. Of the 103 participants, 35.92% (95% CI: 26.50–45.34) indicated that they take care of animals either at their homes, work (abattoir), or their farms, while 47.57% (95% CI: 37.76–57.38) of the respondents mentioned that they also slaughter animals at home. Furthermore, 10.68% (95% CI: 4.61–16.75) and 25.24% (95% CI: 16.71–33.77) of the respondents indicated consumption of undercooked or raw meat and unpasteurised milk, respectively. Results showed that most (95.15%) of the participants spend most of their time (5–7 days) at work. Additionally, most of the abattoir workers demonstrated an excellent practice of prevention of zoonotic TB, as 94.17% (95% CI: 89.57–98.77) had personal protective gear, and 95.15% (95% CI: 90.92–99.37) wore their personal protective equipment (PPE) when handling carcasses (Table 3).

**TABLE 3:** Practices that may promote infection with tuberculosis-causing mycobacterial species in abattoir workers in the workplace and outside activities and environment.

Variable	Category	%	Frequency		95% CI
			*n*	*N*	
Do you take care of animals at home, work, or farm?	Yes	35.92	32	103	26.50 to 45.34
If yes, which types of animals?	Livestock	67.74	21	103	−44.50 to 111.20
	Pets	25.81	8	103	-
	Wildlife	6.45	2	103	-
How many days do you work in the abattoir per week?	1–2 days per week	2.91	3	103	−99.60 to 166.20
	3–4 days per week	1.94	2	103	-
	5–7 days per week	95.15	98	103	-
Do you consume unpasteurised milk?	Yes	25.24	26	103	16.71 to 33.77
Do you consume uncooked or undercooked meat?	Yes	10.68	11	103	4.61 to 16.75
Do you slaughter animals at home?	Yes	47.57	49	103	37.76 to 57.38
Do you have personal protective gear?	Yes	94.17	97	103	89.57 to 98.77
Do you wear personal protective gear?	Yes	95.15	98	103	90.92 to 99.37
Have you been vaccinated against TB?	Yes	50.00	46	103	39.59 to 60.41

TB, tuberculosis; CI, confidence interval.

### Signs and symptoms experienced by workers while working in the abattoirs and associated age

Of the 103 respondents, 79.6% reported experiencing symptoms associated with TB, including fever, cold, loss of appetite, cough, body pain, night sweats, and weaknesses. A statistically significant association between age groups and respondents with TB was observed (*p* = 0.009), with workers aged 36–50 years (57.14%) more prone to TB infection (Table 4). Interestingly, this was the age group with the highest number of respondents who consumed unpasteurised milk.

**TABLE 4:** Age and history of TB suffering and knowledge of zoonotic implications among abattoir workers.

Variable	Frequency (Yes)	Frequency (%)	*p*-value
**Have you ever been sick with TB?**			
**Age group (years)**	-	-	0.009
18–25	0	0.00	-
26–35	0	0.00	-
36–50	4	57.14	-
51–60	3	42.86	-
**Humans can infect animals?**			
**Age group (years)**	-	-	0.030
18–25	4	9.76	-
26–35	16	39.02	-
36–50	11	26.83	-
51–60	10	24.39	-

TB, tuberculosis.

## Discussion

Most of the respondents in this study were male (84.47%, *n* = 87/103) and young adults aged 26–35 years old (*n* = 43/103). This characteristic was also observed in a previous study, where most respondents were young adults. This is probably because of young adults being able-bodied and possessing the physical strength required in abattoir settings (Ismaila, Rahman & Saliluddin 2015). This study found that over 80% of the respondents knew about TB, a frequency much higher than in a previous study conducted in Nigeria. For example, a survey by Kachalla and co-workers found that 53.2% of abattoir workers at two abattoirs in Abuja, Nigeria, had a fair knowledge of the disease (Kachalla et al. 2016). In South Africa, it was previously reported that participants’ bovine TB knowledge was 61% (Marange, Morar-Leather & Fasina 2020), which is lower than that observed in this study. A possible reason for the higher levels of knowledge of TB observed in this study may be partly because of the fact that the research team made both oral and written presentations on zoonotic diseases during earlier visits to the same abattoirs to collect samples from slaughtered livestock, on TB (Mareledwane et al. 2021), brucellosis (Kolo et al. 2019), Q fever and toxoplasmosis (Mangena et al. 2023), and leptospirosis (Dogonyaro et al. 2020). Hence, such interventions are crucial for providing information that can help prevent diseases. It is important to note that a low level of knowledge poses a considerable public health risk, as abattoir workers are in a high-risk occupation for diseases (Dogonyaro et al. 2020). It is pertinent to mention that although the frequency of knowledge of TB in the abattoir workers we studied was high (88.35%), the use of PPE was equally high at 95.15%. It was however, revealed that among this cohort of abattoir workers, there was a gap in their knowledge of aspects of TB such as its transmission. This is because their awareness was comparatively low regarding their ability to transmit the disease to animals (44.6%) or animals’ ability to transmit it to them (45.7%). Future studies may need to explore the definition of knowledge in questionnaire-based studies. Datiko and colleagues found that a lack of transmission pattern awareness is a risk factor, as it leads to disease transmission. In this study, several factors associated with zoonotic TB transmission, such as caring for animals at home and at work, consuming unpasteurised milk, eating uncooked or raw meat, and slaughtering animals at home, were identified as promoting TB transmission (Datiko et al. 2019). Ingestion of raw meat, unpasteurised milk, and contaminated droplets from an infected animal is considered the main route of transmission of TB from animal to human (Sa’idu et al. 2015). This study showed that 25.24% of respondents consumed unpasteurised milk and 10.68% consumed undercooked or raw meat, a concerning finding. This finding is comparable to a study by Mohammed and colleagues in Nigeria, who reported that 24.1% of participants consumed unpasteurised milk (Mohammed et al. 2019). The same study, however, reported a significantly higher percentage of participants (31.2%) who consumed undercooked meat than our study. In Africa, there is an increase in the custom of eating raw meat and unpasteurised milk (Sichewo et al. 2020; Van Helden & Michel 2019) because of cultural and traditional beliefs about their nutritional benefits (Deneke et al. 2022), which is a very unfortunate situation.

Over 40% of abattoir workers in this study slaughter animals at home, which has been identified as an unsafe slaughtering practice. This practice has been among the factors found in some African countries that enhance the transmission of zoonotic TB (Swai, Schoonman & Daborn 2010). Our study revealed that abattoir workers in the age group between 36 years and 50 years old were the most involved in slaughtering livestock at home, hence prone to zoonotic TB infection because of close contact with animals that may be infected (Melaku, Sharma & Alemie 2013).

There was a statistically significant (*p* = 0.030) difference in the frequency of workers who knew that humans could transmit TB to animals, and this observation was made at the highest frequency in the age group between 26 years and 35 years old (39.02%) compared to 9.76% for workers between the ages of 18 years and 25 years old. These findings were consistent with previous studies that showed that older age was associated with greater knowledge, as older age groups have more work experience than younger ones (Adesokan et al. 2013).

It is also relevant to observe that the differences in the frequency of abattoir workers who previously had symptoms associated with TB were statistically significant (*p* = 0.009), with 57.14% of workers aged 36–50 years. A total of 18.2% of the workers highlighted that they had a family member previously diagnosed with TB. Respondents who know the signs and symptoms of TB and its transmission are expected to be more likely to practise preventive measures to reduce exposure to the disease (Adebanjo 2011). Other studies have demonstrated that knowledge of TB transmission leads to lower disease prevalence in communities (Sreeramareddy, Harsha Kumar & Arokiasamy 2013; Tschopp et al. 2015).

A limitation of the study was our failure to analyse gender-related percentage frequencies of exposure risk to TB in abattoirs and home environments. It is recommended that this be included in future studies.

## Conclusion

The study found that a high percentage (88.35%) of the abattoir workers had knowledge of bovine TB, and 95.15% wore PPE, which augurs well for reducing exposure to the pathogens that cause the disease within the abattoirs in Gauteng province, South Africa. However, the finding that a relatively low proportion of abattoir workers were aware that they could transmit TB to animals (44.6%) and that they could acquire the disease from animals (45.7%) suggests that they may know about the disease but little about its mode of transmission. It is therefore important to emphasise this in future awareness programmes. Considering the risk of exposure to abattoir workers outside the work environment, it is of zoonotic significance that 10.68% of abattoir workers consume uncooked meat, 25.24% drink unpasteurised milk and 47.5% slaughter animals at home, practices that could increase exposure to *Mycobacterium* spp. including *M. bovis*. Because abattoir workers are in a high-disease-risk occupation, health authorities, together with abattoir management, should regularly educate them not only about zoonotic TB transmission but also about other zoonotic diseases, such as brucellosis and leptospirosis.
